# Microbiome analysis reveals gut bacterial alterations in adult Tibetan pigs with diarrhea

**DOI:** 10.3389/fmicb.2025.1524727

**Published:** 2025-06-30

**Authors:** Zhoulin Wu, Xiaoyu Li, Yan Wang, Jiamin Zhang, Lili Ji, Ling Gan

**Affiliations:** ^1^Meat Processing Key Laboratory of Sichuan Province, College of Food and Biological Engineering, Chengdu University, Chengdu, China; ^2^Animal Breeding and Genetics Key Laboratory of Sichuan Province, Sichuan Animal Science Academy, Chengdu, China; ^3^College of Veterinary Medicine, Southwest University, Chongqing, China

**Keywords:** Tibetan pigs, diarrhea, gut microbiota, 16S rRNA, biomarker

## Abstract

Diarrhea is a significant ailment that causes heavy economic losses in the pig industry. The Tibetan pig is a native Chinese breed that is unique to high-altitude regions and displays strong disease resistance. However, scientific research on the structural characteristics of the gut microbiota and key genera associated with diarrhea in Tibetan pigs is still scarce, especially those involving adult Tibetan pigs. In this study, fresh fecal samples from diarrheic (case, *N* = 9) and healthy adult Tibetan pigs (control, *N* = 10) were collected and sequenced using 16S rRNA gene sequencing. Our results revealed that the gut microbial community of the case pigs exhibited lower alpha diversities but higher intragroup variability in microbiota composition. The genera *Treponema* and *Prevotellaceae*_UCG-001 were underrepresented in the pigs, serving as hallmarks of diarrhea, while *Lactobacillus*, *Escherichia-Shigella*, and *Muribaculaceae* showed increased abundance. Moreover, the genera *Lactobacillus* and *Ignatzschineria* were significantly enriched biomarkers in the case pigs. Notably, these changes were not consistent with those observed in Tibetan piglets and other commercial pigs. Furthermore, the decreased abundance of *Treponema* in the diarrheic pigs indicated that this disease was associated with a high-fiber diet and environmental adaptability. The differentially enriched pathways in the case and control pigs further revealed that gut dysbiosis exacerbated immune and inflammatory responses to promote the development of diarrhea. In conclusion, this study characterized the distribution of gut microbiota composition in adult Tibetan pigs with different health status, which may enhance our understanding of the role of the gut microbiota in intestinal issues.

## 1 Introduction

The Tibetan pig is a unique native Chinese breed that mainly lives on the Qinghai-Tibetan plateau. It is characterized by high adaptability to harsh environments, such as hypoxia, severe coldness, intense ultraviolet radiation, and rough feeding (Yang et al., 2011; Yang et al., 2017). These characteristics contribute to its strong environment and disease resistance, which are reflected in its genome and its unique intestinal flora (Zhao et al., 2023). Like all animals, Tibetan pigs have a complex intestinal microbiota community that includes bacteria, archaea, protozoa, and eukaryotic organisms, populated by hundreds to thousands of different microbes (Liu et al., 2021). A stable gut microbiota is a prerequisite for various normal metabolic activities and overall host health (Lynch and Pedersen, 2016), while gut dysbiosis has often been associated with digestive ailments such as diarrhea, weight loss, and irritable bowel syndrome in both pigs and humans (Qi et al., 2021; Kim et al., 2023).

Diarrhea is a common disease that seriously threatens animal husbandry, causing productivity losses and even death (Li B. et al., 2019; Panah et al., 2021). It can affect pigs of all ages, especially piglets during weaning (Ruiz et al., 2016). Generally, diarrhea in neonatal pigs can be fatal, with diarrheic infections accounting for up to 49% of deaths (Morris et al., 2002), while it usually leads to loss of appetite, submissive behavior, and reduced fertility in adult swine. Many factors, including genetic and environmental influences—such as toxicity, nutrition, stress, and infectious and non-infectious factors can cause this disease. Among these, infectious factors, including bacteria, parasites, and viruses, contribute to its high incidence (Lee et al., 2018; Lazov et al., 2022). The gut microbiota plays a direct role in the development of gut diseases such as diarrhea and inflammatory bowel disease due to its function in defending against pathogenic invaders (Li Y. et al., 2021; Wang et al., 2023). It is well established that this disease can disrupt the host’s gut microbiota, regardless of the causative factor. Extensive research has highlighted the disruption of gut flora caused by diarrhea across various livestock species, such as lambs (Kong et al., 2019), dairy calves (Kim et al., 2021; Cendron et al., 2020), goats (Cheng et al., 2022), commercial piglets (Sun et al., 2019), and early-weaned Tibetan piglets (Qi et al., 2021; Kong et al., 2022). Despite this, there is a lack of knowledge regarding the gut microbiota in adult Tibetan pigs with the problem of diarrhea.

On the other hand, previous studies have tried to better understand the differences in the intestinal microbes of Tibetan pigs at different growth stages (Jiang et al., 2018). Furthermore, the composition and diversity of the intestinal microbiota in swine change depending on age and breed (He et al., 2023; Yang et al., 2022). Based on previous findings, it is hypothesized that diarrhea alters the gut microbiota structure and function in adult Tibetan pigs, potentially in ways different from those observed in Tibetan piglets or commercial piglets. To test the hypothesis, the study aimed to evaluate the effects of diarrhea on the gut microbiota of adult Tibetan pigs (1–1.5 years).

## 2 Materials and methods

### 2.1 Feces sampling

In this study, female Tibetan pigs (1–1.5 years old), inhabiting the Ganzi Tibetan Autonomous Prefecture, were used for sample acquisition (Kangding city, Sichuan, China; 100°E, 28°N, approximately 3,500 m above sea level). All animals grazed on the same pasture during the day and were housed in a livestock shed at night, where each pig was offered ~1 kg of corn kernels. The health status of the pigs was assessed by trained veterinarians. Diarrheic pigs (case) were identified based on symptoms including dehydration, reduced feed and water intake, and feces that were thin, unformed, and gray or gray-white watery, lasting more than 2 days. In contrast, the feces of the healthy pigs (control) were granular or stripe-shaped. Fresh fecal samples from the case group were collected on day 2 after symptom onset, and the age-matched controls were also sampled. From January to August 2021, a subset of 19 fresh fecal samples (one sample per animal, ~8 g each) were collected from the herd, including nine samples from the case group (*N* = 9), and 10 from the control group (*N* = 10). Each sample was collected in a sterile 50 mL plastic tube, labeled, and transferred to a −80°C freezer until DNA extraction.

### 2.2 DNA isolation and sequencing

Total bacterial genomic DNA was extracted from each thawed sample using a stool DNA isolation kit (Qiagen, Shanghai, China). Genomic DNA concentration assessment was carried out using a NanoDrop ND1000 spectrophotometer (NanoDrop Technologies, Montchanin, DE, United States), and its purity and integrity were checked by 2% gel electrophoresis. Afterwards, 30 ng of the DNA was used to amplify the hypervariable V3–V4 regions of the 16S rRNA gene using primers 338F (5′-ACTCCTACGGGAGGCAGCA-3′) and 806R (5′-GGACTACHVGGGTWTCTAAT-3′). PCR was performed with 25 cycles of 94°C (30 s), 55°C (30 s), 72°C (60 s), and 72°C (10 min). After this, 2% agarose gel electrophoresis was used to extract the amplicon products. Subsequently, the qualified amplicons were quantified for sequencing library construction, and 2 × 250 bp paired-end sequencing of the qualified library was performed on the Illumina HiSeq 2500 platform.

### 2.3 Bioinformatics and data analysis

Sequence data analysis was performed as previously reported (Wu et al., 2024), using the open-source bioinformatics tool Quantitative Insights into Microbial Ecology 2 (QIIME2) (Bolyen et al., 2019). Briefly, the pyrosequencing reads were demultiplexed and assigned to their original samples based on their unique barcode sequences. Next, the Trimmomatic software (Bolger and Giorgi, 2014) was used to screen the qualified reads. Afterward, primer sequences were trimmed from the sequences using Cutadapt (1.9.1) (Martin, 2011), resulting in high-quality target reads. *De novo* operational taxonomic units (OTUs) were then clustered at a 97% sequence identity threshold using QIIME2, with redundant sequences removed during the process. Representative OTUs were aligned through the DEBLUR program (Amir et al., 2017). Then, taxonomic information was obtained by aligning them to the SILVA reference database (v138). We used the Chao1 and Shannon diversity indices to evaluate alpha diversity, and statistical differences were assessed using the Kruskal–Wallis test. Beta diversity was calculated using the Bray–Curtis dissimilarity and the unweighted UniFrac metric within the QIIME2 platform. Cluster analysis was visualized using principal coordinates analysis (PCoA). Statistical analyses were performed using R (v4.1.3). Linear discriminant analysis (LDA) effect size (LEfSe) was conducted to identify specific taxa and biomarkers that were differentially abundant between the diarrheic and healthy pigs, using an LDA score threshold of 4.0 at the genus level and above (Segata et al., 2011). A *p*-value of <0.05 was considered statistically significant, and the results were presented as means ± SD. The software PICRUSt2 (v1.7.3) was utilized to predict the functional profiles of significantly different taxa associated with the animals’ health status (Douglas et al., 2020). Pathway predictions were made using the KEGG database.

## 3 Results

### 3.1 Analysis of sequencing data and taxonomy

**Figure 1:**
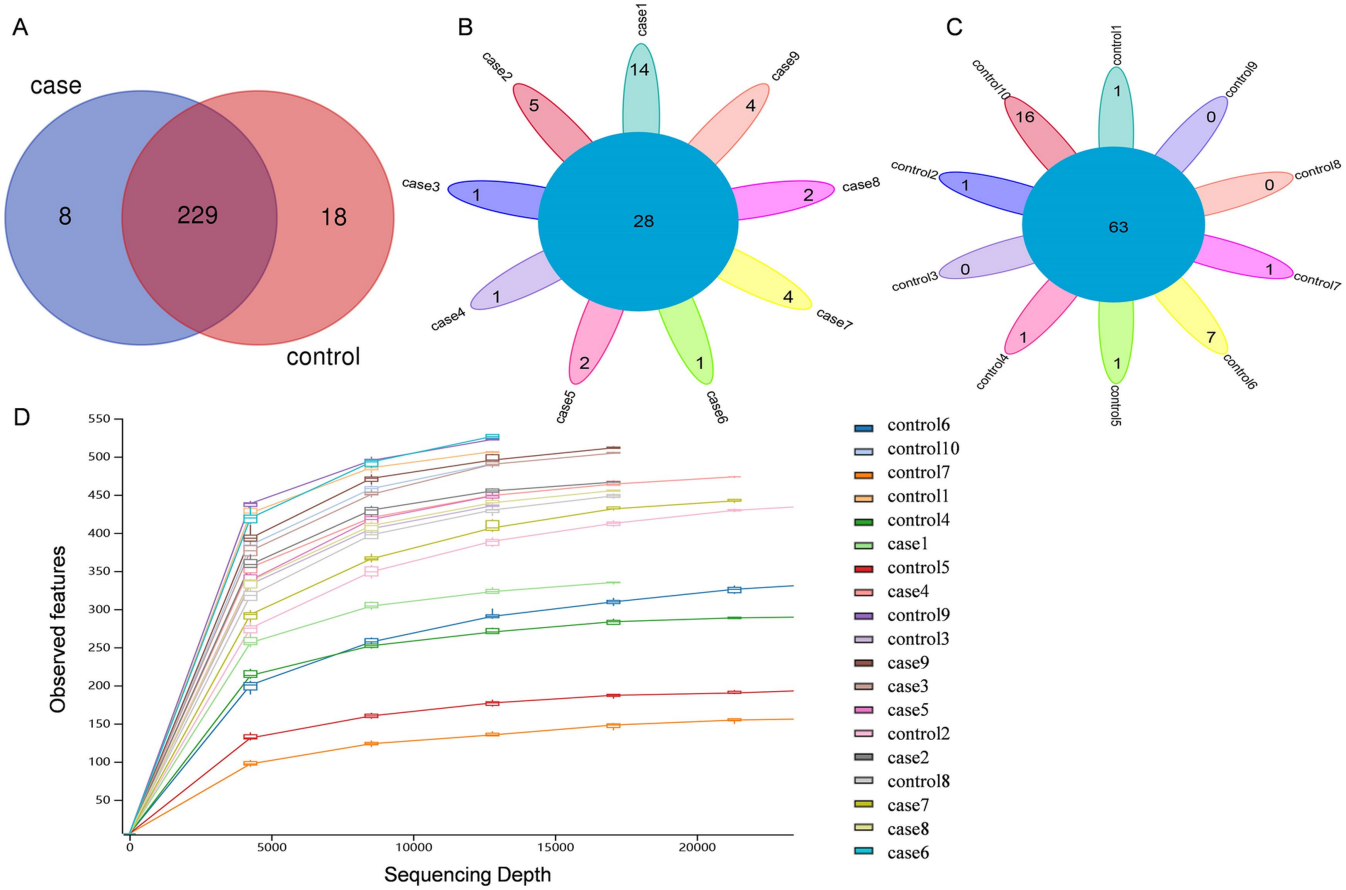
Venn diagrams and rarefaction curves of the case and control Tibetan pigs. Venn diagrams showing the OTU distribution between the case and control pigs **(A)**. Venn diagrams showing the core OTUs in the samples of the case group **(B)** and control group **(C)**. Alpha rarefaction curves of the observed features **(D)**.

After the quality control of the raw data, a total of 1,520,136 high-quality paired-end reads with an average of 80,007 per fecal sample were obtained from all the 19 animals. As shown in Figure 1A, these sequences were assigned to 255 bacterial OTUs (237 in the case group and 247 in the control group) at ≥97% similarity. Of these, 229 OTUs were shared among all pigs, accounting for ~89.80% of the total OTUs. Venn diagrams demonstrated that there were 28 common OTUs among the case pigs (Figure 1B) and 63 common OTUs among the control pigs (Figure 1C). Subsequently, a rarefaction analysis of the observed features was performed to assess sequencing depth. It was observed that as sequencing depth increased, the curves for each sample gradually flattened and plateaued, indicating that the sequencing depth was adequate in all samples for further analysis (Figure 1D).

### 3.2 Microbial community diversity of the Tibetan pigs in the different groups

**Figure 2:**
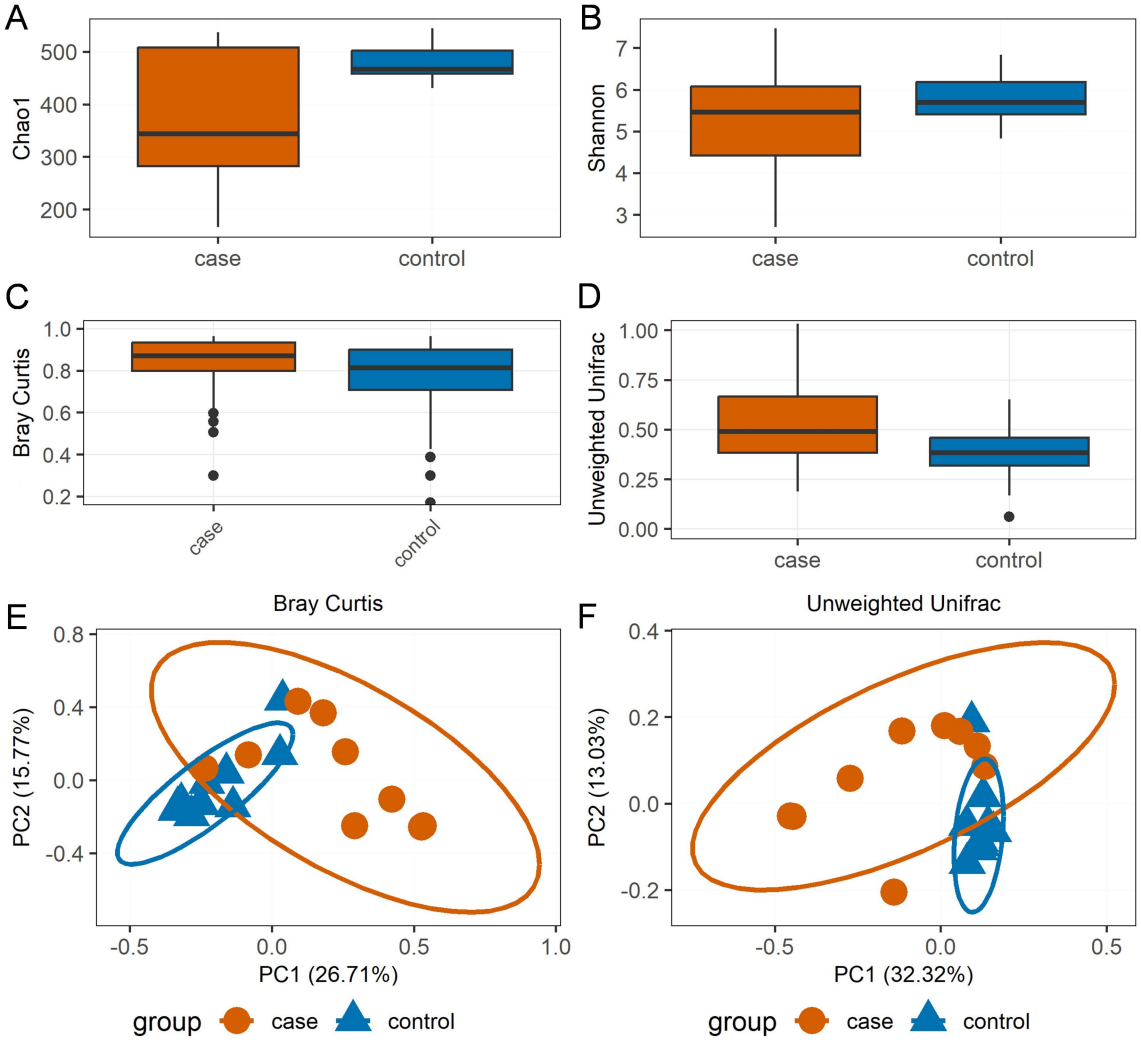
Bacterial community diversity distinguishes the case and control Tibetan pigs. Chao1 **(A)** and Shannon **(B)** alpha diversity indices by sampling group; boxplot showing the beta diversity distances calculated using the Bray–Curtis dissimilarity **(C)** and unweighted UniFrac distances **(D)**. The bottom of each box represents the first quartile, the top of each box represents the third quartile, and the middle line inside the box represents the median value. Scatterplot from principal coordinates analysis (PCoA) based on the Bray–Curtis dissimilarity **(E)** and unweighted UniFrac distances **(F)**.

In terms of species richness and diversity, as measured by the Chao1 (Figure 2A) and Shannon indices (Figure 2B), the case Tibetan pigs showed lower alpha diversity compared to the control animals. However, according to the boxplot of the Bray–Curtis dissimilarity (Figure 2C) and unweighted UniFrac distances (Figure 2D), the case Tibetan pigs showed increased beta diversity compared to the control animals. The PCoA results based on both Bray–Curtis dissimilarity (Figure 2E) and unweighted UniFrac distances (Figure 2F) showed that the bacterial communities and structures of the case and control pigs ranged from completely different to partially similar. Interestingly, the samples from the control group formed more homogeneous clusters, while the case samples displayed greater heterogeneity.

### 3.3 Composition of the microbial community structure in the different groups

**Figure 3:**
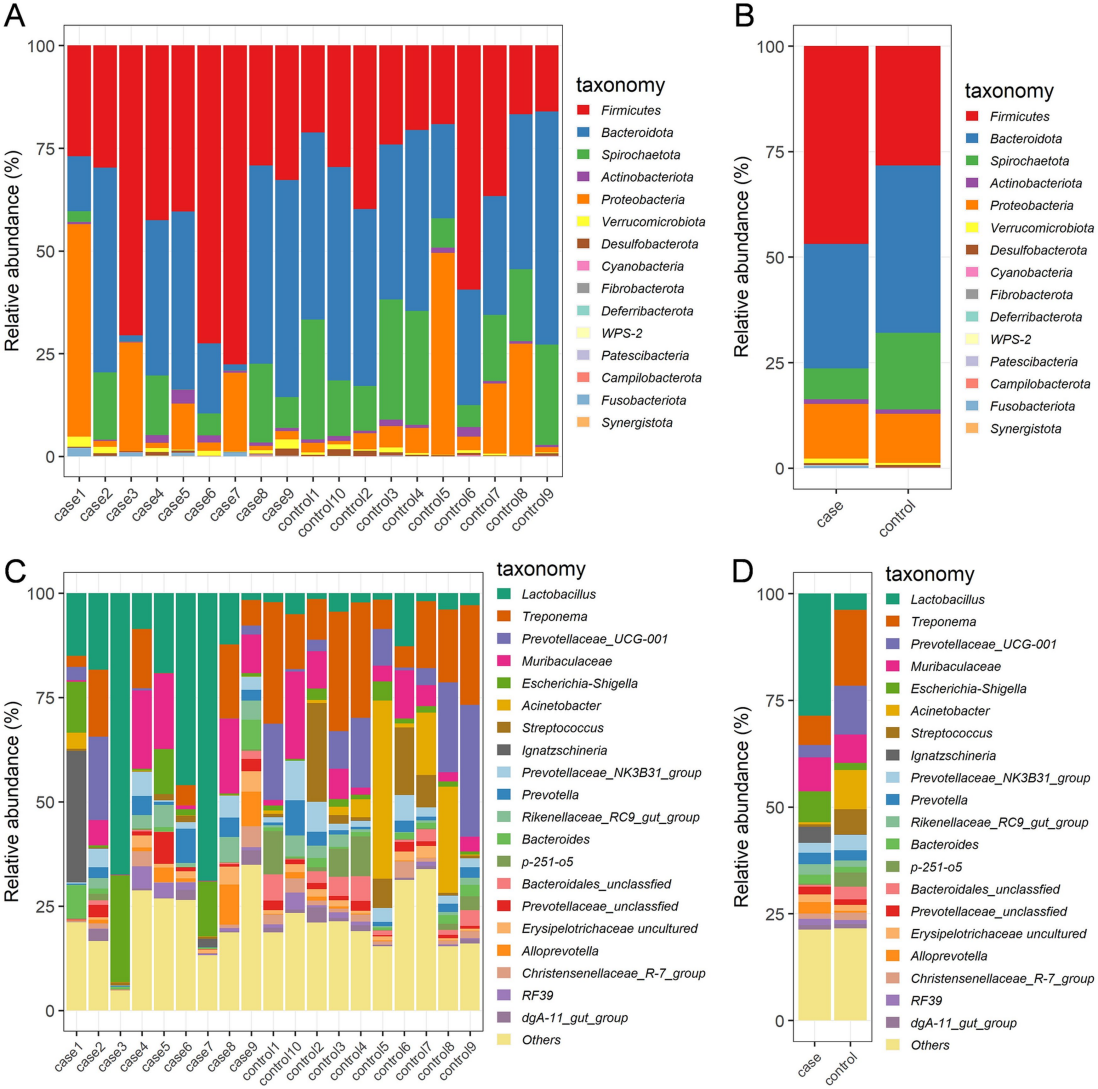
Gut bacterial phyla and genera in the case and control Tibetan pigs. Each bar shows the distribution of bacterial phyla in each individual **(A)** and both groups **(B)**; the distribution of bacterial genera (top 20) in each individual **(C)** and both groups **(D)**.

**Figure 4:**
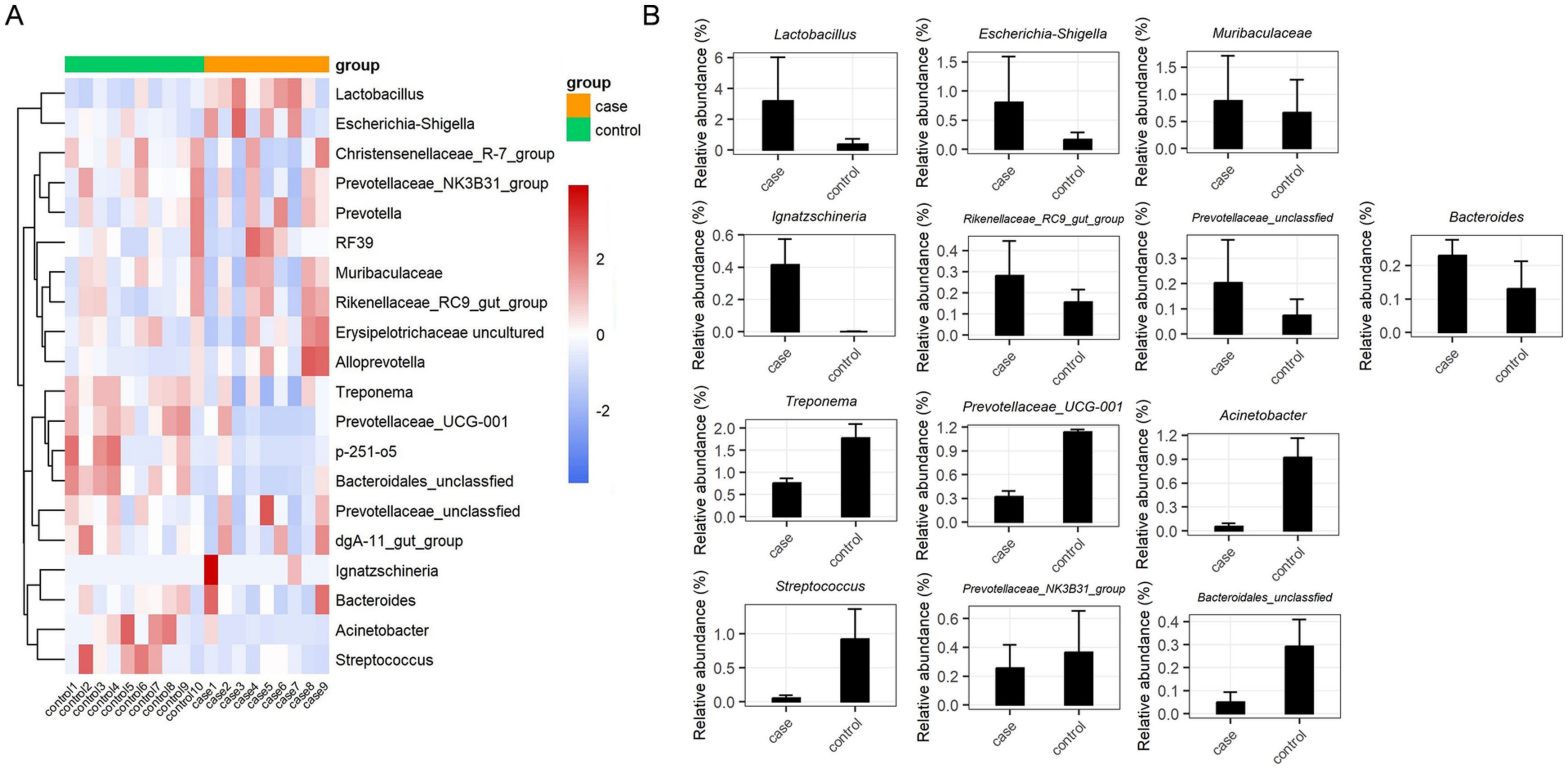
Significant dysbiotic genera in the gut bacterial composition of the case and control Tibetan pigs. Heat map comparing the top 20 genera in the gut bacterial community of each individual **(A)**, and summary of the 13 important taxa that were differentially expressed between the case and control samples **(B)**.

The relative abundances of dominant bacterial communities at the taxonomical levels of the phylum and genus were analyzed based on the taxonomic assignment of all samples. A total of 15 phyla were identified in the 19 samples, of which Firmicutes (37.63%), Bacteroidota (34.57%), Spirochaetota (12.71%), and Proteobacteria (12.28%) were the four dominant phyla (Figure 3A), collectively accounting for approximately 97% of the sequences. Of note, the case group had a significantly higher relative abundance of Firmicutes compared to the control group, while the abundance of Spirochaetota was decreased in the case animals as compared to the controls (Figure 3B). In addition, 189 genera were identified in these samples, and the 20 most expressed genera of the gut flora are presented in Figures 3C,D. The most prevalent bacteria included *Lactobacillus* (16.24%) and *Treponema* (12.32%), followed by *Muribaculaceae* (7.29%), *Prevotellaceae*_UCG-001 (7.14%), *Acinetobacter* (4.86%), and *Escherichia-Shigella* (4.46%). Moreover, the distribution patterns of the bacterial genera in each sample are shown in the heat map (Figure 4A). In addition, the abundance of *Lactobacillus* (3.18%), *Escherichia-Shigella* (0.81%), *Muribaculaceae* (0.88%), *Ignatzschineria* (0.42%), *Rikenellaceae_RC9_gut_group* (0.28%), unclassified *Prevotellaceae* (0.20%), and *Bacteroides* (0.23%) tended to increase in the case pigs compared to the controls (0.39, 0.17, 0.86, 0.01, 0.16, 0.13, and 0.13%, respectively). Conversely, the relative abundance of *Treponema* (0.76%), *Prevotellaceae*_UCG-001 (0.32%), *Acinetobacter* (0.05%), *Streptococcus* (0.06%), *Prevotellaceae_NK3B31_group* (0.26%), and unclassified *Bacteroidales* (0.05%) was lower in the case pigs than in the control animals (1.78, 1.14, 0.92, 0.60, 0.37, and 0.29%, respectively) (Figure 4B).

**Figure 5:**
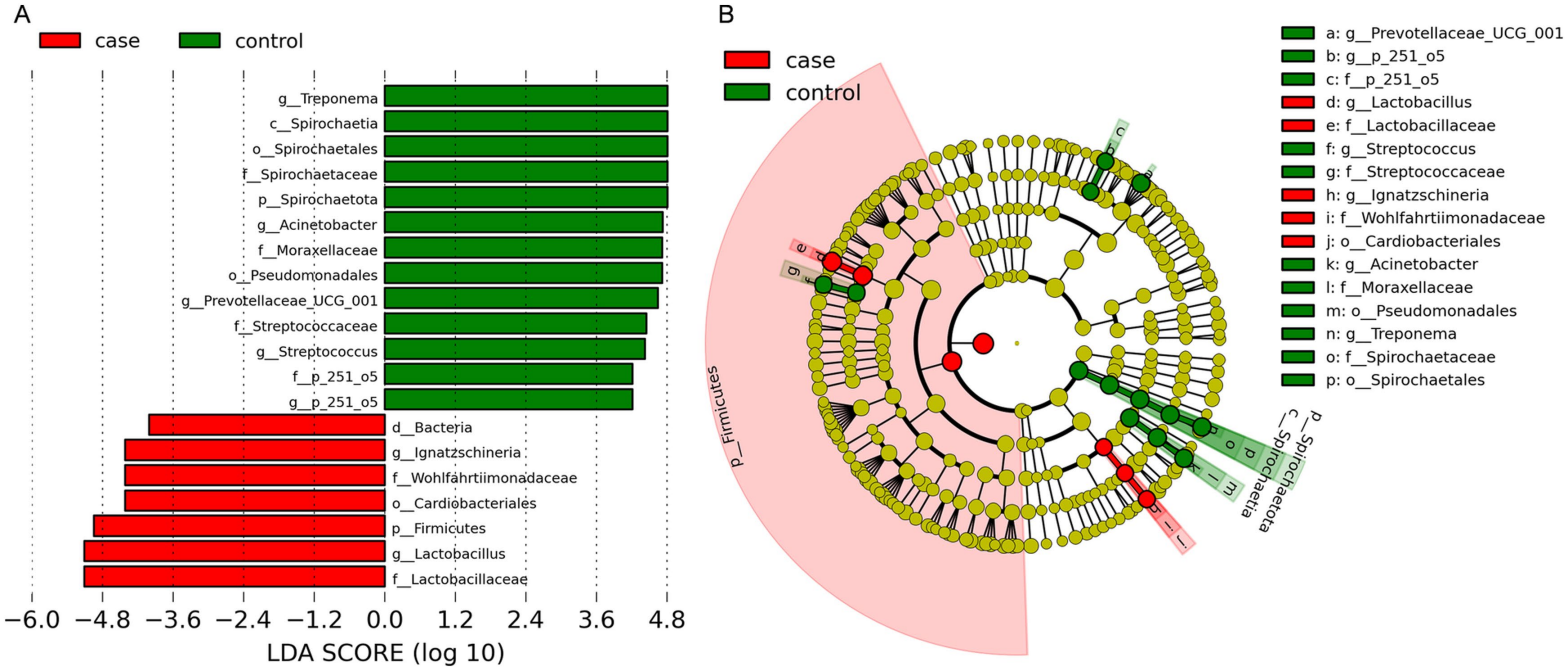
Differential phylotypes between the case and control Tibetan pigs on the basis of linear discriminant analysis effect size (LEfSe) analysis. Histogram of the linear discriminant analysis (LDA) scores indicating bacterial groups significantly enriched in the case (red) or control (green) samples **(A)**. Cladogram illustrating statistically and biologically consistent taxonomic differences between the two groups **(B)**.

We performed LEfSe analysis to identify biomarkers of the diarrheic pigs and found a total of 19 biomarkers with LDA values >4, including seven genera, six families, three orders, one class, and two phyla (Figure 5A). The genera *Lactobacillus* and *Ignatzschineria* were significantly enriched biomarkers in the case pigs, while *Treponema*, *Acinetobacter*, *Prevotellaceae*_UCG-001, *Streptococcus*, and *p-251-o5* were significantly enriched biomarkers in the control pigs (Figure 5A). Of note, all these genera were among the top 20 most abundant taxa (Figure 3D). The relative abundance of *Lactobacillus* was 16.24%, and it was significantly associated with diarrhea in the control group. Furthermore, the cladogram represents the phylogenetic distribution of microorganisms related to health status (Figure 5B).

### 3.4 Predicted functional differences in the gut microbiota between the case and control Tibetan pigs

**Figure 6:**
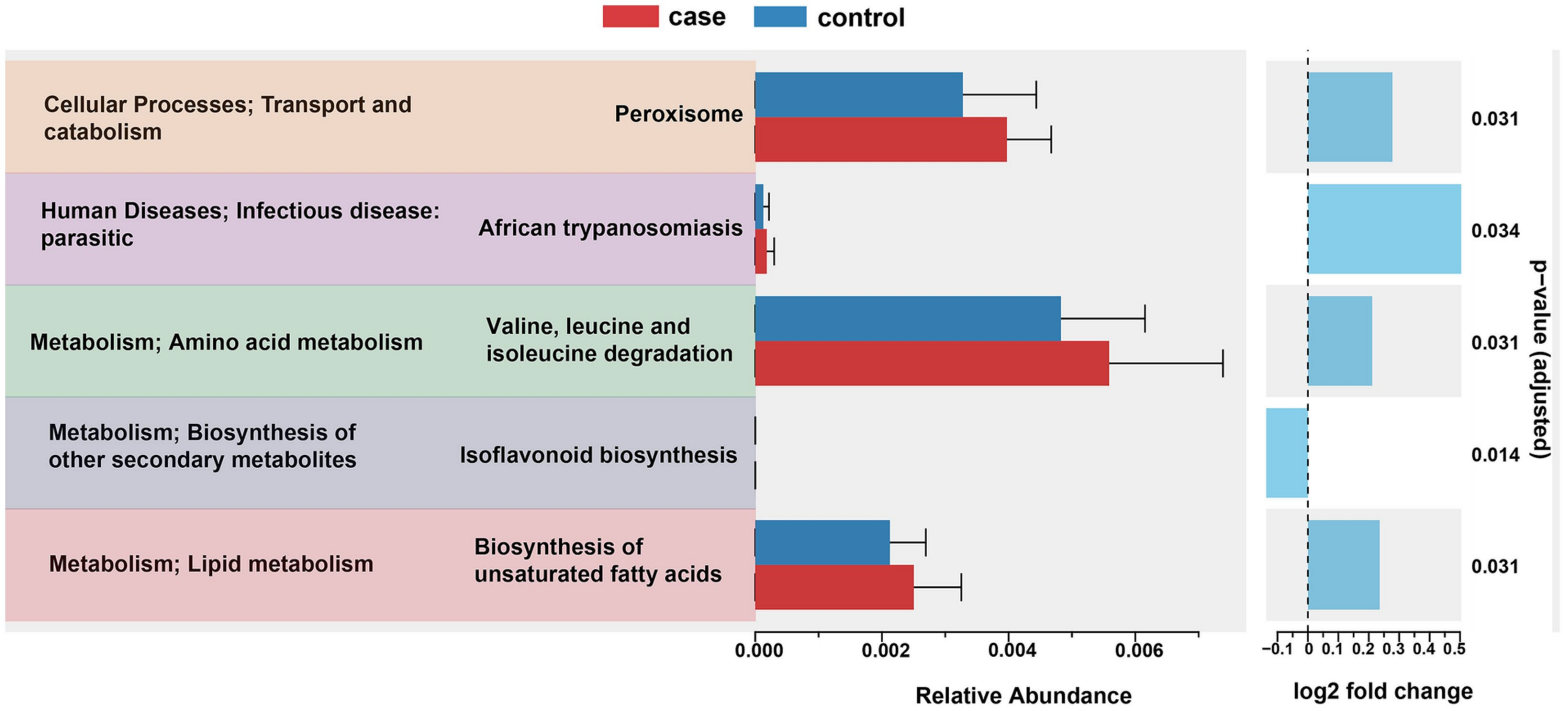
PICRUSt2 predictions of functional differences in the gut microbiota between the case and control Tibetan pigs. Significant differences in the KEGG pathways were identified using ANOVA with a corrected *p*-value threshold of <0.05.

To further predict the functional composition of the gut microbiome related to the phenotype of the case and control pigs, we used PICRTSt2 to infer the global functions of the gut microbiota based on the KEGG annotation database. As illustrated in Figure 6, a total of 10 affiliated KEGG pathways between the case and control pigs were detected to achieve statistically significant differences at an adjusted *p*-value of <0.05. Notably, the pathways related to peroxisome, African trypanosomiasis, valine, leucine and isoleucine degradation, and the biosynthesis of unsaturated fatty acids were significantly enriched in the case group. On the other hand, the pathway of isoflavonoid biosynthesis was significantly enriched in the control group.

## 4 Discussion

Diarrhea is widely prevalent across different animal species and is considered an important factor leading to the disruption of production and causing a large number of deaths. To date, a wide range of factors such as pathogenic agents, weaning, dietary composition, growth stages, physiological status, and environmental conditions have been linked to diarrhea (Langel et al., 2020; Liu et al., 2022; Mai et al., 2020). Although there is considerable evidence suggesting that intestinal function and microbiome could be one of the reasons for diarrhea (Li Y. et al., 2019), how the taxonomic and functional architecture of the gut flora changes during diarrhea in adult Tibetan pigs remains largely unknown. To address this knowledge gap, we comprehensively evaluated the gut microbiome in fresh fecal samples using 16S rRNA sequencing. Comparing the characteristics and predicted functional potential of bacterial taxa between the case and control pigs may provide a foundation for guiding the design of future preventative measures against diarrhea in adult Tibetan pigs.

Consistent with the findings of a previously reported study (He et al., 2023), the phylum Firmicutes was the most predominant, along with enrichment of Bacteroidota and Spirochaetota. Importantly, Firmicutes possess many healthy gut bacteria that contribute to improving the intestinal environment, with some also providing protection against pathogenic invasion (Li A. et al., 2019). Interestingly, diarrhea was associated with an increased relative abundance of Firmicutes and a decreased relative abundance of Spirochaetota, which is in line with the findings of Wang et al. (2023). However, this contrasts with the results of a study on commercial diarrheic piglets, where the phylum Proteobacteria was enriched during diarrheic episodes, while Bacteroidetes showed a decline (Sun et al., 2019). Generally, gut microbiota changes at the genus level reflect pathogenic infections and may indicate gut dysbiosis. The abundance of *Treponema*, *Escherichia-Shigella*, *Sphaerochaeta*, *Slackia*, and *Staphylococcus* has been associated with diarrhea in piglets (Zhu et al., 2024). Our results revealed that the abundance of *Lactobacillus*, *Escherichia-Shigella*, *Muribaculaceae*, *Ignatzschineria*, and *Bacteroides* was significantly higher in the diarrheic samples, while the abundance of *Treponema*, *Prevotellaceae*_UCG-001, *Acinetobacter*, *Streptococcus*, *Prevotellaceae_NK3B31_group*, and unclassified *Bacteroidales* was decreased. Thus, we speculate that the observed changes in relative abundance at both the phylum and genus levels in diarrheic pigs may be influenced by breed and age.

*Lactobacillus* bacteria possess several probiotic properties and play positive roles in antipathogenic activity (Yang et al., 2015), antioxidant activity (Wang et al., 2013), and immune system (Suda et al., 2014). A low abundance of *Lactobacillus* is considered a potential indicator of gastrointestinal problems in piglets (Gryaznova et al., 2022). In previous studies, it was found that diarrheic commercial piglets (Sun et al., 2019; Mach et al., 2015) and Tibetan piglets (Qi et al., 2021) showed a lower abundance of *Lactobacillus*. However, our data revealed a higher abundance of *Lactobacillus* in the diarrheic pigs. The difference may be due to variations in animal breeds and harsh feeding conditions, but further investigation is needed to confirm these factors. In addition, we found a much higher abundance of *Escherichia-Shigella* in the pigs with diarrhea. Several members belonging to the *Escherichia-Shigella* group are known to play key roles in diarrhea (Bin et al., 2018). Our data suggest that these bacteria may play an important role in causing diarrhea in adult Tibetan pigs. Other species such as *Treponema* and *Prevotellaceae*_UCG-001 were present at low abundance in the case pigs. Notably, *Treponema* has been reported to be enriched in the gut microbiota of high-altitude swine and the hunter-gatherer Hadza people, where it contributes to environmental fitness and adaptation to a high-fiber diet (Schnorr et al., 2014). Our findings revealed that the diarrheic animals had reduced *Treponema* in their gut. Interestingly, a similar result was observed in another livestock species on the Qinghai-Tibetan plateau, where the abundance of the genus *Treponema* was decreased in diarrheic yaks (Wu et al., 2022). We hypothesize that diarrhea may alter the gut microbiota associated with adaptation to a high-fiber diet and environmental conditions, but this requires further investigation.

In this study, the cause of diarrhea was not taken into consideration; instead, we focused on comparing the characteristics of microbiota composition between groups. Gut microbial diversity and community structure are positively related to intestinal function (Bui et al., 2020), with alpha and beta diversity metrics commonly used to assess the classification and variation of microbial communities among subjects (Patel et al., 2019). Previous research has shown that higher gut microbial abundance supports the maintenance of intestinal homeostasis and physiological function (Bui et al., 2020). Conversely, in this study, we found lower alpha diversity in the diarrheic samples, consistent with findings in other animal species including rats (Ma et al., 2020), piglets (He et al., 2020), horses (Li et al., 2022), yak (Wu et al., 2022), and giraffes (Li A. et al., 2021), indicating gut microbial dysbiosis. Moreover, PCoA based on Bray–Curtis dissimilarity and unweighted UniFrac distances (Figures 2E,F) was performed and revealed that the bacterial communities in each group ranged from completely different to partially similar. Furthermore, the bacterial functional predictions using PICRUSt2 indicated that the gut bacteria of the diarrheic pigs were more robustly associated with pathways related to peroxisome, amino acid metabolism, and the biosynthesis of unsaturated fatty acids. This suggests that changes in gut microbiota composition may influence the host’s immune function. For instance, peroxisomes help eliminate microbial infections by modulating canonical innate immunity pathways through ROS signaling (Di Cara et al., 2018). Furthermore, unsaturated fatty acids possess anti-inflammatory properties and suppress inflammatory responses (Morrison and Preston, 2016). Together, we conclude that gut dysbiosis exacerbated immune and inflammatory responses to promote the development of diarrhea.

## 5 Conclusion

The diarrheic adult Tibetan pigs showed lower alpha diversity and higher beta diversity of the gut microbiota than the control animals. The levels of specific bacteria changed noticeably with health status. The phylum Firmicutes in the diarrheic pigs was much more abundant than in the control animals. At the genus level, *Lactobacillus*, *Escherichia-Shigella*, *Muribaculaceae*, *Ignatzschineria*, *Rikenellaceae*_RC9_gut_group, unclassified *Prevotellaceae*, and *Bacteroides* were significantly more abundant in the diarrheic pigs. Among them, *Lactobacillus* and *Ignatzschineria* were significantly enriched biomarkers of this disease. Moreover, diarrhea may have disrupted the gut microbiota balance associated with a high-fiber diet and environmental adaptability. This dysbiosis exacerbated immune and inflammatory responses to promote the development of diarrhea.

## Data Availability

The datasets presented in this study can be found in online repositories. The names of the repository/repositories and accession number(s) can be found at: https://www.ncbi.nlm.nih.gov/, PRJNA1183544.
